# Recurrence of Decay at the Cervix

**Published:** 1895-06

**Authors:** J. Foster Flagg


ARTICLE IV.

### RECURRENCE OF DECAY AT THE CERVIX.

PROF. J. FOSTER FLAGG.

You all admit that recurrence of decay at the cervical border gives you the greatest difficulty to surmount, and as yet you have not reached the couse nor the remedy. It must be admitted that if this one thing can be mastered, we have overcome our most powerful foe.

It is a fact not to be denied that every dentist cries out for some method to prevent recurrence at the cervix. This is positive proof that ever one has hands full of proximal cavities from the cuspid back. Every one must admit that contour fillings have been the only help or partial cure, though it has to be repeated or requires patching.

A case presents where caries has run wild. Not a proximal surface scarcely but is involved. No pulps quite exposed, but threatening. Every tooth has been filled and refilled, and by more than one dentist. Contour has been attempted. Where the fillings of gold remain they are so undermined there is nothing but utter annihilation unless all are removed. The teeth from their loss of proximate surfaces are all out of articulation, which can be best seen by taking an impression and putting the casts in my articulator. Look closely at the cervix, and you will find the root of each so close that no thread can be forced through and the decay is far up under the cervix. Look further and probe for the alveolar process, and not a vestige of it remains for a quarter of an inch up. Look also to the second molar where the first has been extracted, and on that side the process has gone far down and nothing but loose gum tissue remains, and is constantly receding, and wherever a tooth has been lost the process about the cervix absorbs as the body of the jaw absorbs.

In this state of affairs you put on your dam and separator, and you obtain a slight widening, and at once fill permanently the excavated tooth. No attention is paid to the articulation of the teeth. You have no desire to wait, and you rush on headlong to fill and get your pay. The rest of the teeth are lefc without anything in them, till one by one you have had your patients at least twice a week for months, two hours or more at a sitting, till they are exhausted and condemn dentistry, and while you are rushing through to complete every cavity with a filling, you have done nothing to prevent further rapid decay, and pulps become exposed and patients have to suffer.

I know this is the case with nearly every man's practice. I see it every week, and I know from personal contact in conversations with patients of others who have not come for treatment.

You can do better than this, and not only retain your patients, but bridge over time as well as space, and fill at your leisure.

I will take the same mouth just illustrated, and without placing in one single permanent filling of any kind of metal, treat it with pink gutta-percha alone, with a little of the white as a facing, where necessary. I cut out only partially the cavities on one side of the jaw, always exposing every grinding surface where the proximal is gone, and make compound by running all of the cavities in one, seldom leaving any proximal cavity to stand alone, but opening it in the grinding surfaces. This is a cardinal principle with me. There is one surface on border I complete at once, and that is the cervical, so that I never have to touch it again, and this I cut so far up as to not only remove all caries, but where I know the gum and process will grow up and over it. This is finished, and to enable me to do so I forgot to say, I never put on the rubber-dam in any case till I fill permanently, when the cervix is firm and will admit of its adjustment. It is easy to stop the blood with perchloride of iron, creasote, or any styptic.

And now for the further treatment. In all the spaces I have made I place great pieces of pink gutta-percha, and, with no separation between them stuff the whole intervening space, trim and let alone. This I do till every place is filled in. I dismiss the patient, and have him call in three or six months or a year, as I may please; and, as I find the teeth wide enough apart for a plus-contour filling, and the alveolar gum border and process is in perfect health, and the process has grown up to the gutta-percha, then I fill only those that show that they are far enough apart at the cervix to permit a healthy, full process to grow that the gum will have proper substance, and cleave to the root and cover up and over the margin of the filling at the cervix. In this is your future security at the alveolar border.

No one has ever called attention to the difference in width of the proximal spaces at the cervix for the bicuspids and molars. The gutta-percha should remain in till double the width or space is gained between the molars than the bicuspids, on account of the greater size of the molars, where more proximal surface is in contact and no room left for cleansing, unless the spaces are very much greater than normal, and the contour made to suit this issue. Here is where you will say, "You will destroy the articulation and cause greater strain on the filling and the teeth." No, you are mistaken. When the whole of these proximal surfaces are filled with the semi-elastic stopping, and the act of mastication set up, the teeth that at first are out of the normal position, and only touch on part of their crown surfaces, are now allowed to readjust themselves; as the gutta-percha will give where the greatest pressure is brought to bear, and where least resistance is offered, no change occurs. I am not mistaken in this. Try it.

This method is a test for any further treatment, which, if needed, can so easily be done. It permits of weeks, months and years before the permanent filling need be introduced. No danger of decay, none of loss of structure from fracture. And, in fact, you can dismiss the patients thus treated with the greatest indifference as to the issue.

Do you ask whether I charge for all this work, and when I send in my bill? I charge for even my thoughts as well as my work. My patients never object to the gutta-percha.

Thus I practice with all; and I am happy in this, knowing that I do far more good, am not troubled about immediate root filling,—fillings falling out,—"conservative treatment of dental pulp." Nor does pyorrhœa ever invade on my domain of original work, because I know the value of articulation, and how to make every tooth perform its individual and collective function, and no improper pressure given it to press or work its life out of it and give rise to the denudation of the peridental membrane: nor is the food ever found pressing up in the cervical border and remaining, nor the cervix so weakened by want of contact with firm alveolar processes, and the gum is left to hug the root at this vital portion so tightly that nothing ever creeps in to cause recurrence.

Any dentist who allows his original patient who follows orders to have pyorrhœa should be sued for damages. See that no food presses on the gum border; see that no tooth is improperly pressed and contorted by false articulation, caused by improper width and contouring; allow no biting of threads, cracking of nuts, biting of ice on one tooth only, or, when a tooth has been lost, see that the articulation is restored, and my word for it gout or no gout, syphilis or disease, phorrhœa will not come, except filth and malaise of one or more teeth.

Gutta-percha used as matrices for gold, amalgam, or oxiphosphate fillings I will not dwell on; you need nothing better. For holding teeth in position after correction where there are cavities in both, I need only mention it. As for assistance on the temporary and permanent teeth, to keep the ligature from slipping down on the cervix by carrying the ligature through it; for fastening pins in roots for crowns; as a medium between crowns and roots to prevent further caries; as a protection to all roots when a gold crown is used; and, in fact, as a factor in our practice, there is nothing to fill its place.—*International.*

